# Effect of a Partially Hydrolysed Whey Infant Formula Supplemented with Starch and *Lactobacillus reuteri* DSM 17938 on Regurgitation and Gastric Motility

**DOI:** 10.3390/nu9111181

**Published:** 2017-10-28

**Authors:** Flavia Indrio, Giuseppe Riezzo, Paola Giordano, Maria Ficarella, Maria Paola Miolla, Silvia Martini, Luigi Corvaglia, Ruggiero Francavilla

**Affiliations:** 1 Department of Pediatrics, University of Bari Aldo Moro, 70125 Bari, Italy; paola.giordano@uniba.it (P.G.); maria.ficarella@hotmail.it (M.F.); mpmiolla@gmail.com (M.P.M.); rfrancavilla@gmail.com (R.F.); 2 Laboratory of Experimental Pathophysiology, National Institute for Digestive Diseases, IRCCS Saverio de Bellis, 70013 Castellana Grotte, Italy; griezzo@gmail.com; 3 Neonatology and Neonatal Intensive Care Unit, Department of Medical and Surgical Sciences (DIMEC), University of Bologna, S. Orsola-Malpighi Hospital, 40138 Bologna, Italy; silvia.martini4@gmail.com (S.M.); luigi.corvaglia@unibo.it (L.C.)

**Keywords:** infant formula, regurgitation, gastric emptying, *Lactobacillus reuteri*, partially hydrolysed whey, starch

## Abstract

Functional regurgitation (FR) is common in early infancy and represents a major drain on healthcare resources. This double-blind, randomized controlled trial investigated the effects of a formula containing partially hydrolysed, 100% whey protein, starch and *Lactobacillus reuteri* (DSM 17938) on gastric emptying rate (GErate) and regurgitation frequency in infants with FR. Enrolled infants were randomly allocated to receive either the test formula or a standard starter formula for four weeks. Ultrasound GErate assessment was performed at baseline (week 0) and at week 4; the number of regurgitations, feed volumes and potential adverse events were recorded in a daily diary. Eighty infants aged four weeks to five months were enrolled; 72 (test group = 37; control group = 35) completed the study. Compared to controls, the test group showed greater percentage changes in GErate (12.3% vs. 9.1%, *p* < 0.01). Mean daily regurgitations decreased from 7.4 (0.8) at week 0 to 2.6 (1.0) at week 4 in the test group and from 7.5 (1.0) to 5.3 (1.0) in controls (between-group difference, *p* < 0.0001). Compared to a standard formula, a starch-thickened partially hydrolysed whey protein formula supplemented with *Lactobacillus reuteri* is more effective in decreasing the frequency of regurgitation and improving GErate, and can be of benefit to infants with FR.

## 1. Introduction

Regurgitation is defined as the backflow of gastric contents into the pharynx or mouth [1]. Due to multiple physiological predisposing factors (i.e., predominantly supine position, liquid meals, loose gastro-oesophageal junction), this condition is particularly common in early infancy: more than 50% of infants between three and four months of age experience daily regurgitation, and about 20% have ≥4 episodes of regurgitation per day [2].

Different from gastro-oesophageal reflux disease (GERD), functional regurgitation (FR) is not associated with troublesome complications such as poor growth, inflammatory esophagitis or respiratory symptoms [3,4]. However, it can become a major cause of parental anxiety [5], leading to an increased number of visits to paediatricians: nearly 1 in 5 parents has sought help for frequent regurgitation [6,7]. Moreover, despite their favourable prognosis, infants with FR not infrequently undergo extensive interventions, such as multiple dietary changes and pharmacological treatments, which may or may not be of benefit [8].

The most important non-pharmacological approaches for infants with FR are parental reassurance, avoidance of overfeeding and dietary interventions [3,9]. The use of anti-reflux formulas thickened with starch, guar gum, or locust bean gum may reduce the number of regurgitation episodes [10,11]. Partially hydrolysed protein formulas have also been shown to decrease the volume and frequency of regurgitation in infants [12]; among the possible mechanisms of action, an improvement of gastric emptying has been proposed. Probiotic supplementation with *Lactobacillus reuteri* has also been associated with an improved gastric motility in both animal and human studies [13,14]. In a study from 2014, we demonstrated the efficacy of oral supplementation with this probiotic strain in preventing FR in healthy term newborns [15]. Nevertheless, the effectiveness of the combination of the above therapeutic approaches on reflux frequency and gastric motility has not been evaluated yet. Given that abnormalities of one or more of the three physiologic processes—namely, oesophageal motility, lower oesophageal sphincter function, and gastric motility—can contribute to FR [4], the aim of the present study was to evaluate the efficacy of a formula containing partially hydrolysed whey protein, additional starch, and the probiotic *L. reuteri* in reducing regurgitation frequency and improving gastric emptying in infants with FR.

## 2. Methods

### 2.1. Study Population

This randomized, double-blind, controlled trial was conducted between 1 January 2014 and 28 February 2015 in the Paediatric Gastroenterology Clinic (Department of Paediatrics) of the University of Bari Aldo Moro (Bari, Italy), and in a Paediatric Primary Care Clinic in Naples (Italy).

Infants referred to these clinics were eligible for the study if they were full-term, appropriate for gestational age, exclusively formula-fed, aged between four weeks and five months at the time of recruitment and if they fulfilled the Rome III criteria for FR diagnosis on the basis of retrospective reports from parents or caregivers (i.e., episodes of gastro-oesophageal reflux in the absence of nausea, hematemesis, aspiration, apnea, failure to thrive, difficulty in feeding or swallowing, or abnormal posture for at least one week) [16].

Currently, there is no validated diagnostic questionnaire for infants or toddlers with functional gastrointestinal diseases, unlike that for children and adolescents (Questionnaire on Paediatric Gastrointestinal Symptoms—Rome III Version [QPGS-RIII]) [16,17]. Hence, we translated the Rome III diagnostic criteria for infants and toddlers into a series of questions on signs and symptoms that would be easily understood by parents. Most responses were either Likert-type scales or categorical. Moreover, to assess their own symptoms experienced during the previous four weeks [18], the infants’ parents completed the Gastrointestinal Symptom Rating Scale (GSRS) questionnaire for adults.

Exclusion criteria included congenital malformations, diseases or syndromes that could affect normal growth, cow’s milk protein allergy and any other chronic or allergic disease, treatment with antibiotics, proton pump inhibitors, H2 antagonists or antacids. In order to avoid possible confounding influences, infants receiving probiotic supplementation or formula supplemented with prebiotics and/or probiotics at the time of enrolment were also ruled out.

Infants assessed for eligibility were exclusively formula-fed and received the same enteral feeding regimen and feed volumes until randomization.

This study was conducted in conformity with the principles and regulations of the Helsinki Declaration. Written, informed consent was obtained from the children’s parents/legal guardians, who were fully informed of the nature and purpose of the study. The study protocol was approved by the Ethical Institutional Review Board of Institutional Ethics Committee of Bari University Hospital and is registered in the Protocol Registration System Clinical Trial.gov (ClinicalTrials.gov Identifier: NCT01956682).

### 2.2. Study Design

Enrolled infants were assigned consecutive numbers, starting with the lowest available, and were randomly allocated to the control or experimental group using a computer-generated randomization list. Both parents and physicians were blinded to the group assignment. The control group received a commercially available starter formula that included 70% whey protein and 30% casein, providing 1.85 g of protein per 100 kcal (NAN 1, Nestle Nutrition, Vevey, Switzerland). The test formula was a commercially available (NAN A.R., Nestle Nutrition, Vevey, Switzerland) partially hydrolysed 100% whey formula thickened with starch, providing 1.9 g protein per 100 kcal, and supplemented with a mixture of potato, corn starch (4 g/100 kcal) and *Lactobacillus reuteri* DSM 17938 (2.8 × 10^6^ CFU/g powder). Infants in both groups were fed the assigned formula in standardised amounts according to their weight, age and appetite for four weeks.

### 2.3. Outcome Evaluation

In order to analyse in depth the role of gastric motility in the pathophysiology of FR, the primary aim of this study was the evaluation of gastric emptying rate (GErate). GErate was evaluated at baseline (week 0) and at the end of the study period (week 4) by means of a real-time apparatus (Image Point HX, Hewlett Packard Company, Palo Alto, CA, USA) equipped with a 3.5 MHz linear probe. The probe was placed at the level of the trans-pyloric plane to allow a simultaneous visualization of gastric antrum, superior mesenteric vein and aorta. The antral measurements were taken from the outer profile of the wall. Since the cross section of the gastric antrum, corresponding to the sagittal plane passing through the superior mesenteric vein, is elliptical in shape, its area can be calculated by measuring the longitudinal (L) and anteroposterior (AP) diameters and applying the formula for calculating the area of an ellipse (π L × AP/4) [19]. Antral cross-sectional plane area was used as a proxy for gastric content volume, and measurements were done before and immediately after the end of the test meal (time 0) and at 30, 60, 90, and 120 minutes after the meal. GErate was expressed as the percent reduction in antral cross sectional area at time 0 and 120 min after meal ingestion (GErate = ((antral area time 0 min − antral area 120 min)/antral area time 0) × 100) [20]. The percentage difference between baseline GErate values and those recorded at the end of week 4 was then calculated and used for statistical analysis.

Secondary outcomes were the frequency of regurgitation episodes, growth rates (weight, length, and head circumference, measured at week 0 and 4), and formula intakes. Parents/caregivers were thus instructed to record symptoms (e.g., the number of regurgitations per day, and possible related interventions), feed volumes, administration of any food other than the study formula, and potential adverse events in a structured daily diary, which was returned to the study investigators at the end of the experimental period.

### 2.4. Statistical Analysis

The sample size calculation was based upon the assumption that an improvement in GErate would be expected in 75% of infants receiving the test formula and in 15% of those receiving the control formula. On this basis, a minimum of 30 infants per group was required to achieve an alpha error of 0.05 and a beta error of 0.2. Assuming a dropout rate of 25%, the target enrolment goal was 40 infants per group; recruitment was stopped when this goal was reached.

Data were first analysed using simple descriptive statistics of centrality and dispersion. Regurgitation frequency was calculated as the daily number of regurgitation episodes averaged over the previous seven-day period. To evaluate the distribution of the daily number of regurgitations, a kurtosis test (asymmetry) was performed. The variable was normally distributed at baseline and week 1 but not at other time points, thus at weeks 2, 3 and 4 it was normalized with squaring. Mean values of the normally distributed and normalized variables, including anthropometric parameters, were compared using the Student *t*-test for unpaired samples. An ANOVA model for repeated measures was used to evaluate differences in regurgitation frequency between groups. Differences in gastric emptying features, which were not normally distributed, were evaluated using the Mann–Whitney rank sum test. A per-protocol (PP) analysis was performed.

For all tests, a *p*-value < 0.05 was considered statistically significant. The software package used for the statistical analysis was STATA (STATA version 4.0 Statistical Software, Stata Corporation Houston, TX, USA).

## 3. Results

**Figure 1:**
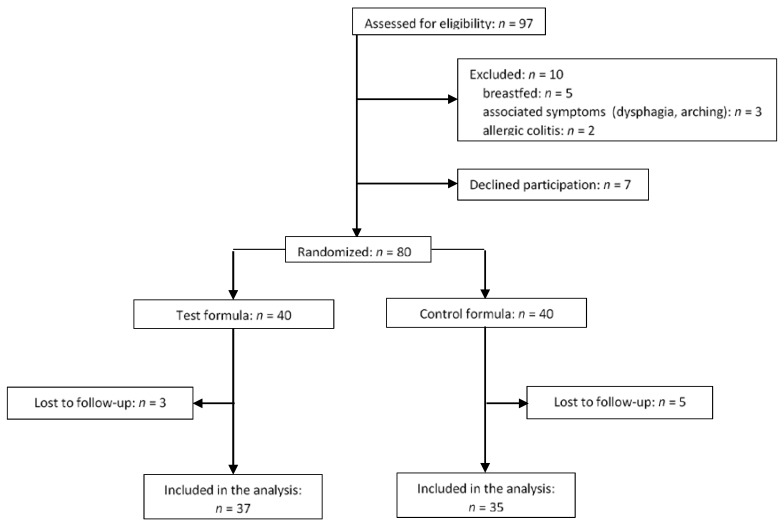
Flow diagram of study enrolment, allocation to the study groups and study dropout.

A total of 80 infants were enrolled and underwent randomization (40 in the test group and 40 in the control group). As shown in the enrolment flow chart (Figure 1), eight of these were lost to follow-up and were thus excluded from the analysis due to incomplete data, whereas 72 completed the trial (37 in the test group and 35 in the control group) and were included in the per-protocol analysis. 

**Figure 2:**
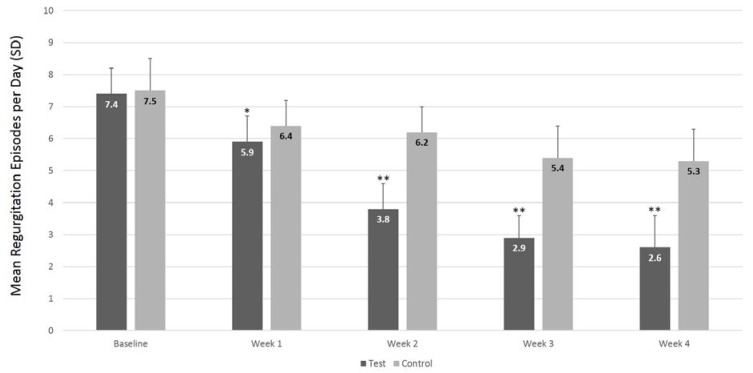
Mean number of daily regurgitation episodes by week in the test and control groups. Repeated measures ANOVA showed a significant difference between groups (*p* < 0.0001) over the entire study period. Means at each time point were compared using Student *t*-test for unpaired samples. * *p* = 0.0094; ** *p* < 0.0001.

At baseline, infants were, on average, 60 days old, weighed 5.6 kg, and had approximately seven episodes of regurgitation per day. No baseline differences in fasting antral areas (Table 1), GErate (test group: median −54.9% [5th percentile = −75.6%, 95th percentile = −44.2%]; control group: median −55.3% [5th percentile = −85.2%, 95th percentile = −44.3%]), age, anthropometric parameters (Table 2) and regurgitation frequency (Figure 2) were seen between the study groups.

With regard to gastric motility parameters (Table 1), at the end of the intervention period the median fasting antral area was significantly reduced in infants receiving the test formula compared to controls (3.5 cm^2^ (5th percentile = 2.0; 95th percentile = 4.6 cm^2^) vs. 4.6 cm^2^ (5th percentile = 2.4; 95th percentile = 6.0), *p* = 0.01). When compared to baseline, median fasting antral areas at week 4 were increased in both groups, consistent with the infants’ growth.

Moreover, infants fed on the test formula showed a significantly higher GErate percentage change between week 0 and week 4 compared to controls (median 12.3% (5th percentile = −3.9%, 95th percentile = 22.0%) vs. 9.1% (5th percentile = −27.0%; 95th percentile = 25.5%), *p* < 0.01). Of note, the 5th percentile of GErate percent change at week 4 was noticeably more negative in the controls compared to the test group (−27% vs. −3.9%), thus suggesting a better gastric motility in the latter.

Infants receiving the test formula showed a significant reduction in the frequency of daily regurgitations compared to the control group (Figure 2). In particular, the mean daily number of regurgitations decreased from 7.4 (standard deviation (SD) = 0.8) at baseline to 2.6 (SD, 1.0; 95% CI = 2.2–2.9) at week 4 in the test group and from 7.5 (SD 1.0) to 5.3 (SD 1.0; 95% CI = 5.0–5.6) in the control group (between-group difference, *p* < 0.0001). No difference in body weight or in the other anthropometric parameters was seen between the two groups at the end of the trial (Table 2). Mean formula intakes were also similar in the test (742 mL/day) and the control (738 mL/day) groups. No adverse events related to both the study formulas were reported.

## 4. Discussion

According to the present results, a starch-thickened, partially hydrolysed starter formula supplemented with the probiotic *L. reuteri* leads to a significant improvement in gastric motility and regurgitation frequency in infants diagnosed with FR.

Functional abnormalities of oesophageal, gastric and enteric nervous system are known to play a contributing role in the multifactorial pathogenesis of regurgitation [21]. By activating the stretch receptors adjacent to the gastro-oesophageal junction, an excessive gastric distension ensuing from an impaired visceral motility has been previously proposed as representing a significant trigger stimulus for transient lower oesophageal sphincter relaxations [22,23], which are among the main pathophysiological mechanisms underlying gastro-oesophageal reflux in the paediatric population [24]. On this basis, the lower fasting antral areas and the higher GErate percentage changes observed in infants fed the test formula might have contributed to the reduction in regurgitation frequency in the present study.

The molecular and physiological pathways through which gut microbiota can influence intestinal motility are far from fully understood. Nevertheless, following recent evidence [25], it is reasonable to postulate that gut neuromuscular apparatus, (i.e., enteric neurons, interstitial cells of Cajal and smooth muscle cells) can act as a potential mediator for the effects that probiotics exert beyond the intestine, on central and autonomic nervous system. The probiotic strain used in this study has been previously shown to significantly improve gastric motility in rodents and preterm infants [13,14,26]; moreover, daily supplementation with *L. reuteri* DSM 17989 has been associated with a lower reported incidence of functional gastrointestinal disorders in term infants at 3 months of life [15]. Similarly, the present results showed an increased GErate delta in infants fed a partially hydrolysed, starch-thickened formula supplemented with *L. reuteri* DSM 17938, thus suggesting that the beneficial effects of this strain can be preserved if added to infant formulas.

When compared to standard formulas, hydrolysed protein formulas have proved to accelerate feeding advancement, to reduce gastrointestinal transit time and to subsequently increase stool frequency [27,28], while their effect on gastric emptying still remains controversial [29,30] and possibly dependent on the extent of hydrolysis [31,32]. According to current literature, partially hydrolysed formulas (PHFs) may offer a useful alternative to intact protein formulas in the dietary management of common functional gastrointestinal symptoms in early infanthood [33,34]. Moreover, a thickened PHF has been shown to significantly reduce the number and volume of regurgitations in infants with FR when compared to a standard, thickened one [12]. The thickened PHF tested in the present study proved to be more effective than a non-thickened, standard formula in reducing the frequency of regurgitations and in improving GErate. However, larger targeted randomized trials are needed to better investigate the exact role of partial protein hydrolysis on regurgitations and gastric emptying in term infants with FR.

To the best of our knowledge, this is the first study aimed at evaluating the combined impact of three different anti-regurgitation dietary strategies, whose effectiveness on gastro-oesophageal reflux has been previously analysed separately; however, a number of limitations need to be acknowledged. First, the data on regurgitation frequency relied on the accuracy of reports from parents and caregivers. Moreover, although the test formula proved to be overall effective, the design of the present study did not allow us to assess the exact contribution of each dietary strategy in improving GErate and reducing the frequency of regurgitations. However, the present results support their synergic beneficial effect on FR. Furthermore, the positive effects observed in this study might be further enhanced by adding other probiotic strains [35], prebiotics or human milk oligosaccharides to the combination tested [36]. Finally, due to the four-week duration of the trial, no conclusions can be drawn on possible long-term effects of the test formula.

As suggested by a recent retrospective study [37], according to which the highest incidence of functional abdominal pain was seen in adolescents who had been affected by colic and regurgitation during the neonatal period, the importance of an appropriate anti-regurgitation treatment in early infancy extends beyond this period. In this regard, further research on the long-term impact of infanthood functional gastrointestinal disorders, focusing not only on their pathogenesis and treatment but also on the development of effective preventive approaches, would be of interest.

## 5. Conclusions

The use of a starch-thickened, partially hydrolysed infant formula supplemented with the probiotic L. reuteri effectively decreases the daily frequency of regurgitation and significantly enhances gastric emptying in infants affected by FR. Targeted studies are needed to shed light on the exact mechanisms through which each component of this formula exerts its beneficial effects, and to evaluate long-term data on efficacy and safety.

## Figures and Tables

**Table 1:** Gastric emptying parameters, expressed as median (5th and 95th percentile) at baseline (week 0) and at the end of the study (week 4) in the test and control groups.

	Test (*n* = 37)		Control (*n* = 35)		*p*-Value
	Week 0	Week 4	Week 0	Week 4	
Fasting antral area, cm^2^	2.7 (2.0, 3.1)	3.5 (2.0–4.6)	2.7 (1.4, 3.1)	4.6 (2.4, 6.0)	0.01 ^a^
GErate percent change from week 0 to week 4, %	12.3 (−3.9, 22.0)		9.1 (−27.0, 25.5)		<0.01 ^b^

GErate: gastric emptying rate. ^a^ *p*-value for between-groups difference at the end of the study. ^b^ *p*-value for between-group difference in GErate percentage change.

**Table 2:** Age and anthropometric measures (mean ± standard deviation) at baseline (week 0) and at the end of the study (week 4) in the test and control groups.

	Test (*n* = 37)		Control (*n* = 35)		*p*-Value ^a^
	Week 0	Week 4	Week 0	Week 4	
Age, days	59 ± 8.2	92 ± 4.3	60 ± 5.3	93 ± 3.3	NS
Weight, g	5590 ± 631	6280 ± 391	5670 ± 739	6320 ± 239	NS
Length, cm	53.7 ± 1.8	58.1 ± 0.8	54.1 ± 1.5	57.7 ± 1.1	NS
Head circumference, cm	40.8 ± 1.3	42.3 ± 0.3	39.7 ± 1.1	41.8 ± 0.7	NS

NS = not significant (*p* > 0.05). ^a^ *p*-values for between-group difference.
